# Structural and Evolutionary Relationships in the Giant Sex Chromosomes of Three *Microtus* Species

**DOI:** 10.3390/genes9010027

**Published:** 2018-01-10

**Authors:** Luz Lamelas, María Arroyo, Francisco Javier Fernández, Juan Alberto Marchal, Antonio Sánchez

**Affiliations:** Department of Experimental Biology, Faculty of Experimental Sciences, University of Jaén, Campus Las Lagunillas s/n, E-23071 Jaén, Spain; luzlamelas85@hotmail.com (L.L.); marroyo@ujaen.es (M.A.); funkymen19@hotmail.com (F.J.F.); jamaor@ujaen.es (J.A.M.)

**Keywords:** *Microtus*, chromosome painting, giant sex chromosomes, sex chromosome heterochromatin, repeated DNA sequences

## Abstract

The genus *Microtus* has high karyotypic diversity. The existence of notable differences in the length of its sex chromosomes contributes to this variation. Variations in size are attributed to the enlargement of their heterochromatin content, which is of such magnitude in some species that they are referred to as “giant sex chromosomes”. Here, we perform an intra- and interspecific analysis of the molecular composition of the heterochromatic blocks in three species with giant sex chromosomes (*Microtus chrotorrhinus*, *M. cabrerae* and *M. agrestis*). Our results show that the heterochromatic content is very similar in both the X and Y chromosomes of *M. chrotorrhinus*, and that their molecular composition is more closely related to the heterochromatic blocks of *M. agrestis* than to the sex heterochromatin of *M. cabrerae*; however, species-specific differences do clearly exist. Interestingly, the euchromatic regions of the X chromosome of all three of these species share a homologous region composed of heterochromatic-related sequences. Our results therefore reinforce the idea that certain similarities in the original organization of these X chromosomes could have facilitated their later enlargement.

## 1. Introduction

The genus *Microtus* (Arvicolinae) includes 65 extant species that arose by rapid radiation over the past 1.2–2 myr [1]. It has one of the highest rates of karyotype diversification of all mammals [2]. *Microtus* karyotypes vary between 2n = 18 in *M. oregoni* [3] to 2n = 62 in *M. duodecimcostatus* and *M. lusitanicus* [4]. However, much of the cytogenetic attention is due to the occurrence of giant sex chromosomes bearing large blocks of constitutive heterochromatin. *M. agrestis* was the first species in which these chromosomes were described, and they are still the largest known X and Y chromosomes, representing about 20% and 12% of their haploid genomes, respectively [5,6,7]. *M. chrotorrhinus* and *M. cabrerae* are also well known for the size of their sex chromosomes [8,9]. These peculiar chromosomes have also been described in two other species (*M. epiroticus* and *M. transcaspicus*); seven other *Microtus* species also show heterochromatin accumulation in their sex chromosomes, albeit on a smaller scale [10,11,12,13].

The giant X chromosomes in *M. chrotorrhinus*, *M. cabrerae*, and *M. agrestis* are large submetacentric biarmed chromosomes. However, the heterochromatin distribution in each differs significantly. In *M. chrotorrhinus*, the heterochromatic block occupies the entire long arm of the X chromosome, while the small arm is completely euchromatic [14]. In *M. cabrerae*, this block occupies the entire short arm and the centromere, and a quarter of the long arm [15,16,17,18], while in *M. agrestis*, this block contains the entire long arm, the centromere, and the most proximal quarter of the short arm [19,20,21]. The Y chromosomes of the three species are similarly divided into a very long arm composed of constitutive heterochromatin and a very small euchromatic short arm [15,16,20,21,22].

Several studies have explored the molecular composition of the sex chromosome heterochromatin in *Microtus* species. These studies show that the heterochromatic content of giant sex chromosomes varies within species. Moreover, diversity in the type of sequences that compose these chromosomes is extraordinary, even in the same species [13]. The list of sequences that are currently known includes satellite DNAs [14,17,22,23,24,25,26,27,28,29], non-tandem complex repeats [7,20,30,31], L1-related retroelements [21,32,33], Long Terminal Repeats (LTRs) retrotransposons [34,35,36], interstitial telomeric sequences (ITSs) [37] and pseudogenes [18,36].

Intra- and interspecific comparisons of the whole content of giant sex chromosomes helps provide a greater understanding of their evolutionary dynamics. Chromosome painting is a very useful approach that has already shed light on this process. Painting analyses with probes from the giant X chromosomes of *M. cabrerae* and *M. agrestis* have shown that the sequences of heterochromatic blocks evolved rapidly and independently in each species [38]. However, the euchromatic content of the X chromosome remains extremely well conserved in all of the analysed species [38,39,40]. Moreover, the giant sex chromosomes in these two species share homologous regions within the euchromatin part of the X and Y chromosomes that are not present in the normal-sized sex chromosomes in other species [40]. This homology could be indicative of a common origin of the giant sex chromosomes and of the process of heterochromatic enlargement that has characterized their evolution [40]. Further comparative analyses with other species would generate a wider evolutionary context.

Here, we provide a detailed analysis of the composition of the heterochromatic blocks of the X and Y chromosomes in *M. chrotorrhinus* using both chromosome painting and fluorescent in-situ hybridization (FISH), with different types of repeated DNAs. We also analyse the chromosome distribution pattern of the painting and repeats probes in two other species with giant sex chromosomes: *M. cabrerae* and *M. agrestis.* The results of these comparative cytogenetic analyses will help improve our knowledge of the evolutionary dynamics of sex chromosomes in *Microtus*.

## 2. Materials and Methods

### 2.1. Chromosome Preparations

We obtained the chromosome preparations from permanent fibroblast male cell lines of three species of the genus *Microtus* (*M. agrestis*, *M. cabrerae*, and *M. chrotorrhinus*) following the procedures described by Neitzel et al. [21].

### 2.2. Chromosome Painting

Two different chromosome painting probes were prepared using the *M. chrotorrhinus* sex chromosomes: one contained the whole acrocentric Y chromosome, which is entirely heterochromatic, while the other included the long arm of the X chromosome (Xq probe), which is also completely heterochromatic. Chromosome microdissection, probe labelling (using Spectrum-Orange dUTP (Abbott Molecular, Abbott Park, IL, USA), hybridization and image capture were performed as described in Marchal et al. [38].

### 2.3. Fluorescent In-Situ Hybridization (FISH)

FISH location of repeated DNA sequences was performed following Fernández et al. [17]. Briefly, probes were labelled with biotin-16-dUTP (Roche Applied Science, Mannheim, Germany) using PCR methods and then detected by avidin-based indirect fluorescence techniques. Four different repeated sequences were analysed: a non-tandem repetitive sequence from *M. agrestis* named pMAHAE2 [7,12]; a fragment from a L1 retrotransposon element from *M. agrestis* named pMAECO14 [21]; telomeric repeats [37]; and the satellite DNA Msat-160, an important component of the pericentromeric heterochromatic of most *Microtus* species [14,17,23,24]. These repeated sequences have been cloned and described for some Arvicolinae species, but have never been analysed and discussed together in the context of sex chromosome organization and evolution.

## 3. Results

### 3.1. Sex Chromosomes Heterochromatin Painting

**Figure 1:**
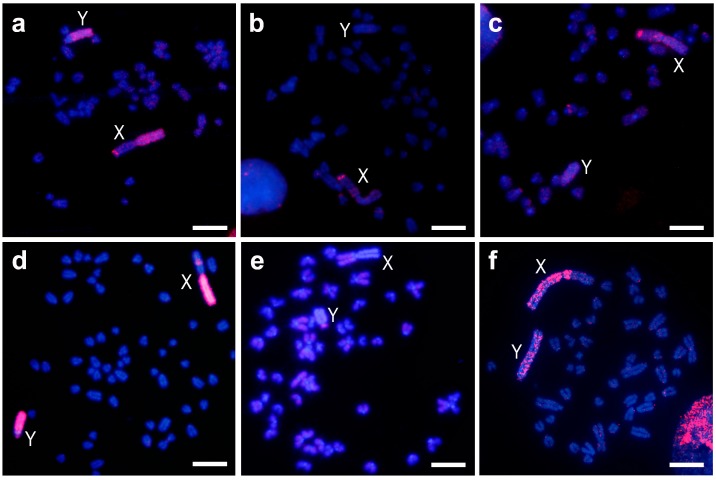
Chromosome painting with the probe of the X chromosome heterochromatin (**a**–**c**) and of the whole Y chromosome (**d**–**f**) of *M. chrotorrhinus* on metaphases of the same species (**a**,**d**), of *M. cabrerae* (**b**,**e**) and of *M. agrestis* (**c**,**f**). Red colour probes hybridizations and blue colour DAPI (4′,6-Diamidine-2′-phenylindole dihydrochloride) staining; X and Y, denoted X and Y chromosomes respectively. Scale bars 4 µm.

The *M. chrotorrhinus* Xq probe hybridized as expected on the entire long heterochromatic arm of the X chromosome, but also on one band of the subtelomeric region of the euchromatic X short arm (Figure 1a). Furthermore, this probe painted most of the heterochromatic Y chromosome long arm. Only a region that included the small arm, the centromere, and a small portion of the long arm proximal to the centromere did not hybridize.

In *M. cabrerae*, this probe hybridized hardly at all on the short arm of the X chromosome, which is entirely heterochromatic; on the other hand, a marked subtelomeric signal was observed on the long arm (almost completely euchromatic, Figure 1b). On the Y chromosome, no hybridization was detected. A similar result was obtained for *M. agrestis* (Figure 1c). Hybridization was limited on the X and Y heterochromatic blocks, and only one strong subtelomeric signal on the euchromatic region of the Xp arm was detected.

The *M. chrotorrhinus* Y chromosome probe hybridized as expected along the whole Y chromosome (Figure 1d). The signal produced was intense throughout almost the entire chromosome, and only a centromere proximal band from the Yp arm appeared less stained. Furthermore, this probe completely painted the heterochromatic long arm of the X chromosome and one interstitial band of the euchromatic Xq arm.

In *M. cabrerae*, this probe only faintly stained the euchromatin of the X and Y chromosomes; their heterochromatic blocks remained unstained (Figure 1e). However, a different result was observed in *M. agrestis* (Figure 1f). In this case, hybridization was clearly observed throughout the heterochromatic blocks of both sex chromosomes, although the signal was not uniformly present. In the euchromatic region of the X chromosome, a less intense signal was observed.

### 3.2. Repetitive Sequences FISH

#### 3.2.1. pMAHAE2

**Figure 2:**
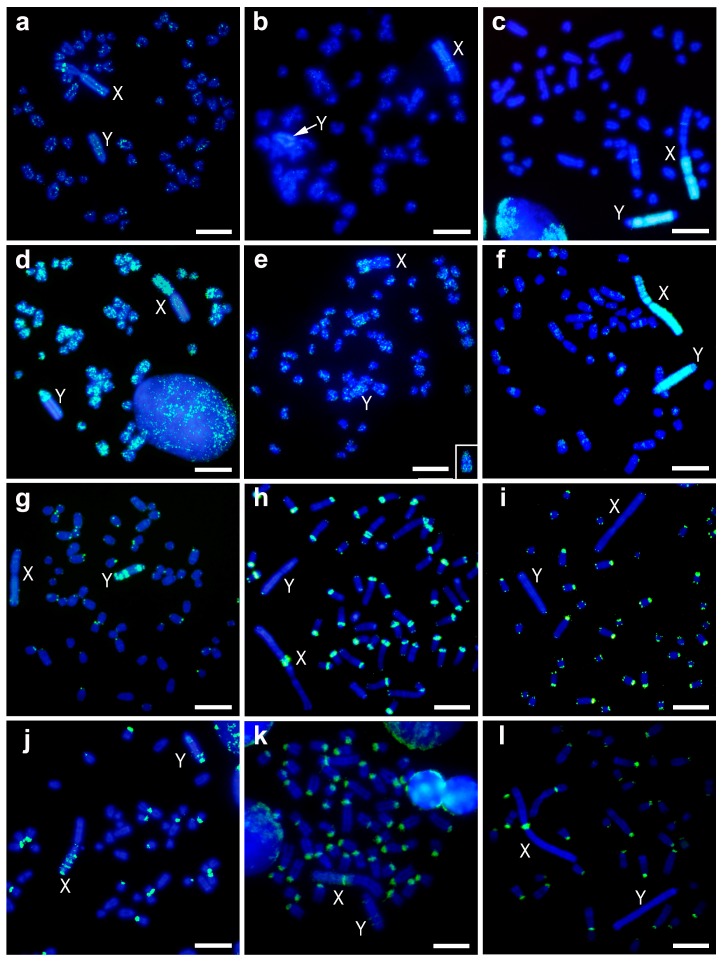
Fluorescent in-situ hybridization (FISH) with different repeat DNA sequences, non-tandem repetitive sequence pMAHAE2 (**a**–**c**), L1 retrotransposon pMAECO14 (**d**–**f**), telomeric sequences (**g**–**i**), and satellite DNA Msat-160 (**j**–**l**). Metaphases of *M. chrotorrhinus* (**a**,**d**,**g**,**j**), *M. cabrerae* (**b**,**e**,**h**,**k**) and *M. agrestis* (**c**,**f**,**i**,**l**). The insert in e is the Y chromosome of another metaphase of *M. cabrerae* hybridized with the same probe. Green colour probes hybridizations and blue colour DAPI staining; X and Y, denoted X and Y chromosomes respectively. Scale bars 4 µm.

The pMAHAE2 repeat DNA sequence was barely present in the heterochromatic blocks of the sex chromosomes in both *M. cabrerae* and *M. chrotorrhinus* (Figure 2a–c). Only a faint dispersed signal was observed, especially in the Y chromosome of the latter species. However, a clear interstitial band was observed in the euchromatic region of the X chromosome in both species (Figure 2a,b). In the case of *M. cabrerae*, this band lies at the interface between the euchromatin and heterochromatin of the X chromosome (Figure 2b). Moreover, pMAHAE2 sequences appeared distributed as spots in some autosomes in *M. cabrerae* and *M. chrotorrhinus* (more intensely in the latter) (Figure 2a,b).

In *M. agrestis*, the FISH pattern obtained for the pMAHAE2 repeat was similar to that originally described (Figure 2c). Although nearly absent from the autosomes (except for one interstitial band), it was highly enriched in the sex chromosomes. Thus, on the X chromosomes this pattern was strongly present in two large heterochromatic regions from the Xq arm and from one interstitial band in the euchromatin. On the Y chromosome, it was heavily amplified throughout most of the heterochromatin but was absent from the terminal region of Yq arm and the pericentromeric region.

#### 3.2.2. pMAECO14

The pMAECO14 repeat sequence produced a signal throughout the chromosomes of *M. cabrerae* and *M. chrotorrhinus* (Figure 2d,e). In particular, it was enriched in the euchromatic regions of the X chromosomes in both species—a to-be-expected genomic distribution for a long interspersed nuclear element 1 (LINE1)-related sequence with mobile capacity. However, this sequence was scarcely present in the heterochromatic blocks of the sex chromosomes. Only one part of the Y chromosome in *M. chrotorrhinus*—which included the Yp arm, the centromere and a centromere-proximal band from Yq—showed a strong signal for this probe (Figure 2d). In *M. agrestis*, the FISH pattern obtained for the pMAECO14 repeat was similar to that originally described and appeared clustered throughout the heterochromatic blocks of both sex chromosomes, but interspersed on the autosomes (Figure 2f).

#### 3.2.3. Telomeric Repeat

As expected, this repeat sequence was located in the telomeric regions of all chromosomes in *M. chrotorrhinus* (Figure 2g). However, in this species telomere repeats were not enriched in the pericentromeric regions of most autosomes, as is the case of *M. cabrerae* and *M. agrestis* (Figure 2h,i). Thus, in these two later species, ITSs are an important component of the centromere heterochromatic fraction. Interestingly, several blocks of ITSs were present throughout the heterochromatin of the Y chromosome in *M. chrotorrhinus* (Figure 2g). This is a different scenario to the one described for the sex chromosomes in *M. agrestis* and *M. cabrerae*. As can be observed in Figure 2h,i, ITSs are lacking from the heterochromatic blocks of both sex chromosomes in *M. agrestis*, while in *M. cabrerae* their presence is limited to a single band at the pericentromeric region of the X chromosome.

#### 3.2.4. Msat-160

The Msat-160 repeat DNA sequence was located in a variable number of pericentromeric regions in the three *Microtus* species analysed, being most abundant in *M. cabrerae* and least abundant in *M. chrotorrhinus*. The pattern of hybridization in sex chromosomes was different in each species. On the *M. chrotorrhinus* X chromosome, several bands were distributed on the heterochromatic long arm and one small signal on the centromere (Figure 2j). On the Y chromosome, however, there were three bands—two large and one small—on the heterochromatic long arm. On the *M. cabrerae* X chromosome, there was a weak signal throughout the entire heterochromatic block and a strong signal in the pericentromeric region (Figure 2k). On the Y chromosome, there are three very faint interstitial bands. With the exception of the strong signal in the pericentromeric regions of the X chromosome, no other signals were observed in *M. agrestis* for both sex chromosomes in either the euchromatin or heterochromatin regions (Figure 2l).

## 4. Discussion

The presence of large blocks of constitutive heterochromatin in both sex chromosomes in *M. chrotorrhinus*, *M. agrestis* and *M. cabrerae* enlarged these chromosomes in comparison to the sex chromosomes of other congeneric species [10,11,15,20]. Here, we provide a detailed analysis of the composition of the heterochromatic blocks of the X and Y chromosomes in *M. chrotorrhinus* using chromosome painting and FISH using several repeated DNAs. From an evolutionary perspective, our analyses are an interspecific comparison of the molecular content of the X and Y giant chromosomes in these three species using the same probes.

Chromosome painting is a very useful method for revealing how different or how similar two particular chromosomes are. The evolutionary dynamics of the giant sex chromosomes in *M. cabrerae* and *M. agrestis* have previously been analysed using this method, and have turned out to be different [38]. In the former, the heterochromatic blocks probably evolved independently in the X and Y chromosomes, thereby giving rise to the different molecular content currently revealed by painting. However, in the latter species, painting analyses have revealed a similar molecular composition in the heterochromatin of both sex chromosomes. In the present study, the painting analyses using either Xq or whole Y probes revealed, firstly, the same result for *M. chrotorrhinus*; that is, both heterochromatic blocks have a very similar composition. This pattern is similar to the one described in *M. agrestis*, and it is therefore likely that the heterochromatic blocks evolved in parallel in both sex chromosomes of this species, as proposed for *M. agrestis* [38].

Our interspecific painting analyses also provide evidence that the composition of the heterochromatic blocks in *M. chrotorrhinus* more strongly resembles those of *M. agrestis* than the sex heterochromatin in *M. cabrerae*. This became evident in particular when the Y heterochromatin of *M. chrotorrhinus* was used as a painting probe. Interestingly, these comparisons also demonstrated that the euchromatic regions of the X chromosome in these three species share a similar band in their subtelomeric regions. This same region appeared stained in *M. cabrerae* when hybridized with painting probes prepared from the giant X chromosomes in *M. cabrerae* and *M. agrestis* [32]. This euchromatic band is likely to be composed of the same repeated sequences in all three species. They do not seem to be only found in the euchromatin, and are also present in the heterochromatic block of the same chromosome, although it remains to be seen how abundant they are. A different interstitial positive signal was observed in the euchromatin of the X chromosome in *M. chrotorrhinus* when hybridized with the whole Y probe. However, this band was not recapitulated in the euchromatin of the giant X chromosome in the other two *Microtus* analysed. From these comparisons, we can conclude that the euchromatic regions of the giant X chromosomes in *Microtus* contain interstitial bands composed of repeated sequences that are also present in their heterochromatic counterparts. While some of these heterochromatic-shared euchromatic bands are species-specific, others appear to be conserved in the giant Xs, which reinforces the idea that certain similarities could exist in the original organization of these X chromosomes that facilitated their subsequent enlargement [40]. This hypothesis requires further investigation, especially in light of the fact that these three species are not phylogenetically closely related. Indeed, while North American *M. chrotorrhinus* always clusters with Nearctic species, the European *M. agrestis* and *M. cabrerae*, endemic to the Iberian Peninsula, are regarded as two ancient independent isolate lineages within the *Microtus* genus [1,41]. In addition, due to its archaic morphological characteristics, some authors include *M. cabrerae* as the only extant species of the genus (or subgenus) *Iberomys* [41,42].

The comparative results obtained using the probe pMAHAE2 add valuable information to our interspecific analyses. pMAHAE2, a 3 kb (GATA)11-positive non-tandem repeated sequence, was originally cloned and described in *M. agrestis* [7]. Interestingly, this repeated DNA is a major component of the heterochromatic blocks of this species, and is nearly absent from other chromosomal regions. However, our analyses show that pMAHAE2 is scarcely detected in the heterochromatic blocks in *M. chrotorrhinus* and *M. cabrerae*. This agrees with previous dot-plot estimations of the copy-number of this sequence (1–2 × 10^4^ in *M. agrestis* and 100 in *M. cabrerae*) [7]. Thus, according to our painting analyses, it is clear that pMAHAE2 is not one of the repeated DNAs that must share heterochromatic blocks in *M. agrestis* and *M. chrotorrhinus*. Remarkably, pMAHAE2 is enriched in one band in the euchromatic region of the X chromosome in both *M. chrotorrhinus* and *M. cabrerae*. This pattern is repeated in several *Microtus* species, and is considered to be an ancestral condition [7]. In conclusion, our data indicate that the pMAHAE2 sequences in *M. chrotorrhinus* and *M. cabrerae*—unlike in *M. agrestis*—retain their original location and are not amplified in the heterochromatic blocks.

Despite the similarities in the appearance of their overall content, certain differences in the molecular composition of the X and Y heterochromatin in *M. chrotorrhinus* were visible after the FISH comparison with some repeated DNAs. This is well illustrated in the ITSs, which are arranged in several interstitial bands in the heterochromatic block of the Y chromosome but are lacking in the X heterochromatin. A contrasting pattern was observed in *M. cabrerae*, where a single interstitial block of ITSs is located in the pericentromeric heterochromatin of the X chromosome, which the Y heterochromatin does not have. The same pattern has been observed in the X chromosomes in *Arvicola sapidus* and *A. terrestris*, and in the Xst1 chromosomes variant in *M. thomasi* [37]. In *A. terrestris*, ITSs also form several blocks in the Y heterochromatin [37], as in our observations of *M. chrotorrhinus*. ITSs are also enriched in the pericentromeric regions of most autosomes in seven *Microtus* species [37]. In summary, ITSs constitute another type of repeated DNA in the arvicolid genome. The enrichment of these sequences in the pericentromeric regions of the chromosomes and in the heterochromatic blocks of the giant sex chromosomes is an unrelated process that has occurred several times during the evolution of these species.

The L1-retrotransposon pMAECO14 also differentiated to some extent the content of the X and Y heterochromatin in *M. chrotorrhinus*. This sequence is nearly absent from the X heterochromatin, but is enriched in the proximal region of the heterochromatic Y chromosome, where it colocalises with ITSs (compare Figure 2d,g). pMAECO14 was originally cloned and described in *M. agrestis*, where it is variably distributed throughout the autosomes and particularly enriched in the heterochromatic blocks [21]. Here, an interspersed autosomal pattern was also observed for this sequence in *M. cabrerae* and *M. chrotorrhinus*, together with a notable accumulation in the euchromatic regions of their X chromosomes. Previous studies have demonstrated that in arvicolid species (as in other mammals), L1-related sequences are not randomly distributed in the genomes or chromosomes [43,44,45]. In the sex chromosomes, they tend to accumulate in the heterochromatic Y chromosomes and inside the euchromatic region of the X chromosomes in both normal-sized and giant chromosomes [32,35].

Differences in the composition of the heterochromatic blocks are also due to satellite DNA sequences. Observed differences in the distribution of Msat160 between X and Y heterochromatin in *M. chrotorrhinus* and *M. cabrerae* have previously been reported [24,25]. Additionally, the repeated DNA sequences Msat-2570 and Msat-21—two tandem repeat DNAs accumulated predominantly in both sex chromosome heterochromatin in *M. chrotorrhinus*—are absent from the sex chromosome heterochromatin in *M. cabrerae* and *M. agrestis* [22,25,26]. The MS2 complex repeat 1194 bp long from sex chromosome heterochromatin in *M. rossiaemeridionalis* is located on the *M. cabrerae* X chromosome heterochromatin but absent from the *M. chrotorrhinus* sex heterochromatin [25].

The two issues that remain to be resolved are: (i) what underlying factors forced heterochromatin to enlarge only in the sex chromosomes of some *Microtus* species and (ii) how this occurred mechanistically. Meiotic exchanges are unlikely to have contributed, since the giant sex chromosomes are asynaptic in most species [46,47]. Unequal sister chromatid exchanges or replication slippage could have favoured heterochromatin amplification independently in each sex chromosome. These two processes could have played a major role during the evolution of *M. cabrerae*, leading to the occurrence of heterochromatic blocks of different composition in each sex chromosome [12,38]. However, in *M. agrestis* and *M. chrotorrhinus* other mechanisms are required that would allow for a homogenization of the heterochromatic sequences between the two sex chromosomes. Translocations of large fragments of DNA triggered by DNA strand breaks occurring in the heterochromatin could have facilitated this process [12]. Whatever the mechanisms, the final result is the appearance of this type of chromosome in only some species. The description of natural populations of one *Microtus* species with extensive sex chromosome polymorphism—in terms of both the size and content of the heterochromatin—provides an interesting evolutionary perspective on this issue [48]. The coexistence of multiple X and Y variants with different heterochromatic content in populations under pressure from genetic drift could lead to the fixation of the largest (or “giant”) chromosomes by random [48].

## 5. Conclusions

In conclusion, our results demonstrate that the heterochromatic content of both the X and Y chromosomes in *M. chrotorrhinus* is very similar. Moreover, their molecular composition is more closely related to that found in the heterochromatic blocks in *M. agrestis* than to the sex heterochromatin in *M. cabrerae*; however, species-specific differences do clearly exist. Our data also reinforce the previously noted heterogeneous content of heterochromatic blocks in *Microtus* sex chromosomes, in both interspecific and intraspecific contexts. Interestingly, the euchromatic regions of the X chromosome in these three species share a homologous region composed of heterochromati-related sequences. Our results therefore reinforce the idea that some particular similarities could exist in the original organization of these X chromosomes, which would have facilitated their enlargement—a process that occurred independently in all three species.
